# Bioremediation and optimization of selenate reduction by a novel *Bacillus cabrialesii* strain (Se1) producing red selenium nanoparticles with potential antibacterial activity

**DOI:** 10.1186/s12866-025-04417-2

**Published:** 2025-11-15

**Authors:** Nagham E. Hossny, Mohamed Ismaeil, Ali M. Saeed

**Affiliations:** Microbiology Department, Faculty of Science, Ain Shams University, Cairo, 11566 Egypt

**Keywords:** Selenate, Se-NPs, Reduction, *Bacillus cabrialesii* and Bioremediation

## Abstract

**Background:**

This study aimed to isolate and identify selenate-reducing bacteria from polluted Egyptian soil. The isolated bacteria could be used to bioremediate contaminated soils and wastewater.

**Materials and methods:**

A potent selenate-reducing bacterium was isolated for optimum production of selenium nanoparticles using a Box–Behnken design (BBD) of the response surface methodology.

**Results:**

A novel selenate-reducing bacterium, designated Se1, was isolated from an industrial effluent soil in Cairo, Egypt. When cultured in enrichment basal medium and then on nutrient agar medium supplemented with 0.945 g L^−1^ sodium selenate, the isolate showed characteristic circular, dark red and shiny colonies. This coloration indicates the reduction of selenate to elemental selenium (Se^0^), with a production yield of 108.8 ± 1.846 μmol. The formation of Se^0^ was confirmed with UV–Vis spectroscopy, which revealed characteristic peaks at 224, 229, and 231 nm. X-ray diffraction (XRD) pattern confirmed the amorphous nature of the synthesized Se^0^ nanoparticles (Se-NPs). Fourier-transform infrared (FTIR) spectroscopy identified diverse absorption peaks within the 400–4000 cm^−1^ range, corresponding to various vibrational modes of chemical bonds, including lipids, proteins, polysaccharides, and functional groups that are present in nanoparticles. Additionally, transmission electron microscopy analysis revealed the presence of Se-NPs within bacterial cells. Based on 16S rRNA gene sequencing, the isolate was identified as *Bacillus cabrialesii* strain Se1 and deposited in GenBank under accession number PP945477. Optimization experiments revealed that the ideal conditions for Se-NPs formation by the isolate were as follows: 3.6 gL^−1^ sodium lactate, pH 7.8, 31°C incubation temperature, 7.6 gL^−1^ selenate concentration, and a ten-day incubation period. Under these conditions, the maximum yield of elemental selenium was 151.311 μmol. The biosynthesized Se-NPs showed potent antibacterial activity against two pathogenic bacteria.

**Conclusion:**

This study presents the first documented evidence of selenate reduction by *Bacillus cabrialesii*, highlighting its potential applications.

## Introduction

Selenium is a hazardous contaminant commonly detected in groundwater, smelting effluents, agricultural and municipal waste, landfill sites, and emissions from power plants. In the. environment, selenium primarily exists in toxic, soluble forms such as selenate [Se(VI)]. However, these can be converted into less harmful, insoluble forms, such as elemental. selenium [Se(0)] and selenide [Se(-II)], which are non-bioavailable and considered nontoxic [73]. Selenate is the most oxidized and bioavailable form of selenium, posing significant health. risks with high exposure. Potential health consequences include headache, nausea, vomiting, metallic taste, and garlic breath odor, as well as damage to the liver, kidneys, and heart. Moreover, repeated exposure can result in pallor, nervousness, and mood changes [12, 58].

The reduction of selenate is an effective method for removing selenium from mine-impacted water, as selenate readily adsorbs to surfaces [5]. Under aerobic conditions, certain bacteria employ lactate as an electron donor and selenate as an electron acceptor, reducing selenate first to selenite and then to insoluble, nontoxic elemental selenium [27]. This elemental selenium is deposited both inside and outside the bacterial cells [6]. Various microorganisms were identified as being capable of reducing selenate and/or selenite, including *Thauera selenatis* [30], *Enterobacter cloacae* (Losi et al. 1997) [35], *Arthrobacter*, *Cephalosporium*, *Citrobacter*, *Corynebacterium*, *Flavobacterium* [22], and *Bacillus selenitireducens* [47]:


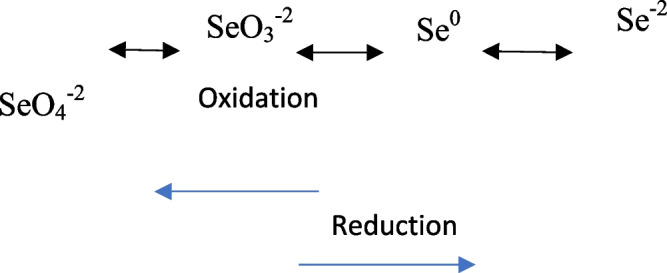


Selenium is an essential trace element necessary for both humans and animals [50]; Baetke et al. 2015 [83]; [76]). It forms part of the amino acid selenocysteine, which is crucial to the catalytic function of various human seleno-proteins and seleno-enzymes, including peroxidases and reductases. Selenium plays a vital role in supporting antioxidant enzyme activity and protecting cells from oxidative damage caused by free radicals (Tran et al. 2011 [16]; [13]. In addition to its biological significance, selenium possesses various industrial applications, including use in semiconductors, photoelectric cells, glass and ceramic pigments, catalysts, and increasingly as selenium nanoparticles (Se-NPs) [79]. However, selenium toxicity is a concern due to its narrow therapeutic window [38]. Consuming more than the recommended daily allowance—0.070 mg/day for men and 0.060 mg/day for women—can be highly toxic to the human body [55, 65].

Se-NPs have demonstrated potential in biological applications due to their antioxidant, antibacterial, antiviral and anticancer properties, making them effective for treating various diseases, including cancer, inflammatory diseases, diabetes and cardiovascular conditions [17, 72]. Additionally, the unique structure of Se-NPs, characterized by a large and uniform pore size, high degradability, and long lifetime, enables them to be combined with other materials for the purification of organic pollutants [41, 45].

The present study aimed to isolate and identify a novel selenate-reducing bacteria (SRB) from polluted soil for the production of elemental Se-NPs. The formation of Se-NPs was confirmed through UV–Vis spectroscopy and Transmission Electron Microscopy (TEM). The study optimized Se-NPs production with the Box–Behnken design (BBD), examining how pH, temperature, and selenate levels affected the process. The antibacterial activity of Se-NPs was tested against two pathogens. Notably, this is the first record of Se-NPs formation by *Bacillus cabrialesii*.

## Materials and methods

### Sampling site and isolation of selenate-reducing bacteria

A polluted soil sample from Anani Canal in Al-Merj, Cairo, Egypt (30.15639° N, 31.37110° E), was collected in a sterile container and kept in an icebox for isolation of selenate-reducing bacteria. The sample was inoculated in enrichment basal medium (EBM) (Cardenas et al., 2021) containing the following:(NH4)_2_SO_4_ (0.225 gL^−1^), K_2_ HPO_4_ (0.225 gL^−1^), KH_2_ PO_4_ (0.225 gL^−1^), NaCl (0.45 gL^−1^), MgSO4.7H2O (0.1 gL^−1^), sodium lactate (2.25 gL^−1^), sodium selenate (0.0945 gL^−1^), vitamin solution (10 mL), and trace element solution (10 mL). The supplied sodium lactate acts as an electron donor, and sodium selenate acts as an electron acceptor. The screw capped tubes were incubated at 30 °C for seven days. Enrichment was repeated three times, and then the final enriched culture was diluted tenfold. Dilutions (100 µL) were subcultured on nutrient agar medium supplemented with 0.0945 gL^−1^ sodium selenate. Subsequently, the plates were incubated at 30 °C for 48 h. After incubation, the red colonies were picked and purified on fresh sterile nutrient agar medium supplemented with 0.0945 gL^−1^ sodium selenate. A deeper red color indicated a higher efficiency of reducing selenate to elemental selenium and a higher production of Se-NPs [2, 62].

### Quantitative assay of elemental selenium produced by bacterial isolates

Selenium production assay was performed according to Cardenas et al.’s method (2021). The selected bacterial isolates were inoculated separately in 15 mL sterile screw-capped tubes containing 10 mL of EBM supplemented with 0.0945 gL^−1^ sodium selenate and then incubated at 30 °C for seven days.

After the incubation period, the bacterial culture with the insoluble red elemental selenium was gently mixed and transferred to 15 mL centrifuge tubes. Bacterial cells with elemental selenium were recovered as a pellet in tubes after centrifugation at 5000 × g and then dissolved in 10 mL of 1 M Na_2_S. Afterward, the sample was centrifuged at 5000 × g to remove bacterial cells. The red-brown supernatant of elemental selenium was measured at 500 nm using the Unico 7200 Spectrophotometer [18, 62].

### Characterization of elemental selenium production

A volume of 100 μl of *Bacillus cabrialesii* strain Se1 was inoculated in a screw-capped test tube containing EBM supplemented with 0.0945 gL^−1^ sodium selenate and incubated at 30 °C for seven days. The culture was sonicated at 50 °C for 15 min and then centrifuged at 5000 × g for 15 min. The pellet was then washed with deionized water, and this process was repeated seven times. Finally, the red Se-NPs were vacuum-dried [1]. The characterization of Se-NPs was performed using UV–Vis spectra, XRD, and FTIR.

### UV–visible absorption spectra

Se-NPs were scanned to obtain UV–visible absorption spectra using Evolution 201 Scan UV–Visible spectrophotometer (Thermo Scientific). Characteristic peaks of elemental selenium were observed between 200 and 300 nm, indicating the presence of elemental selenium [39, 62].

### X‑ray diffraction

A Shimadzu model XRD-6000 was employed to acquire XRD patterns with a range of 2θ from 4° to 90° at room temperature. Cu Kα was employed as a radiation source with a wavelength of λ = 0.15408 nm. Other operating conditions were as follows: scan rate, 8°/min; the operation voltage and current, 40 kV and 30 mA, respectively.

### Fourier‑transform infrared spectroscopy

Se-NPs were characterized by FTIR spectroscopy using a Bruker ALPHA II ATR spectrometer, over a wavenumber range of 400–4000 cm^−1^.

### Transmission electron microscopy (TEM)

TEM was used to investigate bacterial cells and estimate the size of elemental selenium. This was done in the Central Laboratory of the Faculty of Agriculture at Cairo University using a JEM-1200 EX II electron microscope (model JEOL, JEM-1200 Electron Microscope). The formation and size of Se-NPs synthesized by Se1 were measured using TEM, as described by Shahzadi et al. [62].

### Molecular analysis

#### Identification of the selenate-reducing bacterial isolate by 16S rRNA gene sequencing

Total DNA of the bacterial isolate was extracted according to the instruction manual using DNA extraction kits (Thermo, Fisher Scientifics, USA) and stored frozen at– 20 °C until the PCR reaction was carried out. A pair of flanking primers, 8 F (5′-AGAGTTTGATCCTGGCTCAG-3′) and 1542R (5′-AAGGAGGTGATCCAGCCGCA-3′), was used to amplify nearly the complete bacterial 16S rRNA gene. Moreover, 2 μL of the bacterial DNA was used as a template for the PCR reaction. PCR was conducted using Premix Taq (MyTaq, Bioline, UK) according to the instruction manual. PCR was performed using a Genius model FGENO2TD thermal cycler (Techne, England). The PCR conditions were adjusted to 5 min for initial denaturation at 94 °C, followed by 35 cycles of 1 min at 94 °C, 1 min at 54 °C, and 1 min at 72 °C, and finally 10 min at 72 °C for gene amplification.

The PCR amplicons were analyzed on a 1% agarose gel using a 250–10,000 bp DNA ladder to assess product size and purity. The amplified bands were then cleaned using a PCR product purification kit (Thermo, Fisher Scientifics, USA). The obtained PCR product (~ 1500 bp) was then sequenced at the Animal Health Research Institute, Giza, Egypt, using an ABI 3730xl DNA sequencer. The sequence was identified using the BLAST search tool of the National Center for Biotechnology Information (NCBI) and the National Library of Medicine, USA (Madhavi et al. 2012) [19].

Sequence alignment was carried out using ClustalW 1.83 XP, and a phylogenetic tree was constructed with the neighbor-joining method in MEGA version 6. Then, the sequence was submitted using the Bankit tool (NCBI website: www.ncbi.nlm.nih.gov) to obtain the accession number.

### Optimization of selenate reduction and Se-NPs production

A Box-Behnken design (BBD) of the response surface methodology was used to examine the impact of five independent variables on selenate reduction and maximum selenium production. The factors included sodium lactate concentration, growth medium pH, incubation temperature, incubation period, and selenate concentration. According to earlier research, the low and high values of (A) sodium lactate concentration, (B) pH, (C) incubation temperature, (D) incubation period, and (E) sodium selenate concentration were selected [60, 81]; Llamosas et al. 2020)[36] (Table 1).

**Table 1:** The investigated variables with their codes and levels for BBD

Variables	code	Low levels	High levels	Unit
Sodium lactate concentration	A	0.5	5	gL^−1^
pH	B	6	9	-
Incubation temperature	C	25	35	°C
Incubation period	D	2	10	Days
selenate concentration	E	5	25	gL^−1^

Each factor was examined using Minitab Software version 18 at three different levels: − 1, 0, + 1. The model was used to investigate how the various selenate reduction levels interacted and to identify the level of variables that would best lead to the desired maximum Se^0^ production. Moreover, 42 screw-capped tubes containing 10 mL of sterile EBM broth medium inoculated with 100 μL of selected bacterial suspension (10^7^ CFU/mL) were used for the experiments. A spectrophotometer was used to determine the amount of synthesized elemental selenium at 500 nm. All experiments were duplicated, and the mean elemental selenium concentrations were calculated [42, 52, 62].

### Model validation experiment

After model analysis, a validation experiment was the conducted using the ideal process conditions determined by the model response optimizer tool. The experiments were performed in 15 mL screw-capped tubes with 10 mL of sterile broth medium with a starting concentration of 7.6 gL^−1^ sodium selenate. After adjusting the medium’s pH to 7.8, 100 μL of the chosen bacterial suspension (10^7^ CFU/mL) was added, and it was incubated for 10 days at 31 °C with a sodium lactate concentration of 3.6 gL^−1^. The validation experiment was repeated to determine the elemental selenium concentration [60]; Salari et al. 2023) [63].

### Antimicrobial effect of selenium nanoparticles produced by a selected isolate

First,100 μL of the bacterial isolate Se1 was inoculated in a screw-capped test tube containing 10 mL of EBM supplemented with 0.0945 gL^−1^ sodium selenate. Then, it was incubated for seven days at 30°C. To prepare the orange-red Se-NPs, the culture was sonicated at 50 °C for 15 min and then centrifuged at 7,000 rpm for 15 min. Subsequently, the pellet was rinsed with deionized water, and the process was repeated five or seven times, and, finally, the orange-red Se-NPs were vacuum-dried [11, 25].

Antibacterial activity against different pathogenic bacteria was examined using the agar-well diffusion assay [34, 77, 78]. The tested pathogenic bacteria *Staphylococcus aureus* ATCC 29213 and *Pseudomonas aeruginosa* strain E1 (MG847103) were kindly provided by the Microbiology Department, Faculty of Science, Ain Shams University. After placing the samples in Petri dishes pre-inoculated with 100 μL of each pathogenic bacteria suspension, 200 μL of Se-NPs was added to wells cut into nutrient agar medium. The plates were kept at 4 °C for 60 min in the refrigerator to allow diffusion, then incubated at 37 °C for 24 h. For the control, wells were inoculated with de-ionized water. The inhibition zone was measured after the incubation period [11, 25].

### Statistical analysis

An ANOVA test was conducted to examine the collected data. The significance of the model, the coefficient of determination (R^2^) of the developed model, the regression of the examined variables, and the interactions among the variables were all assessed. The findings were used in a polynomial model equation to show the system’s activity and the relationship between the studied variables and selenate reduction (Se^0^ production) (Mallmann et al. 2017 [43] [61].

## Results

### Isolation, screening, and selection of the most promising selenate reducing bacterial isolate

**Fig. 1:**
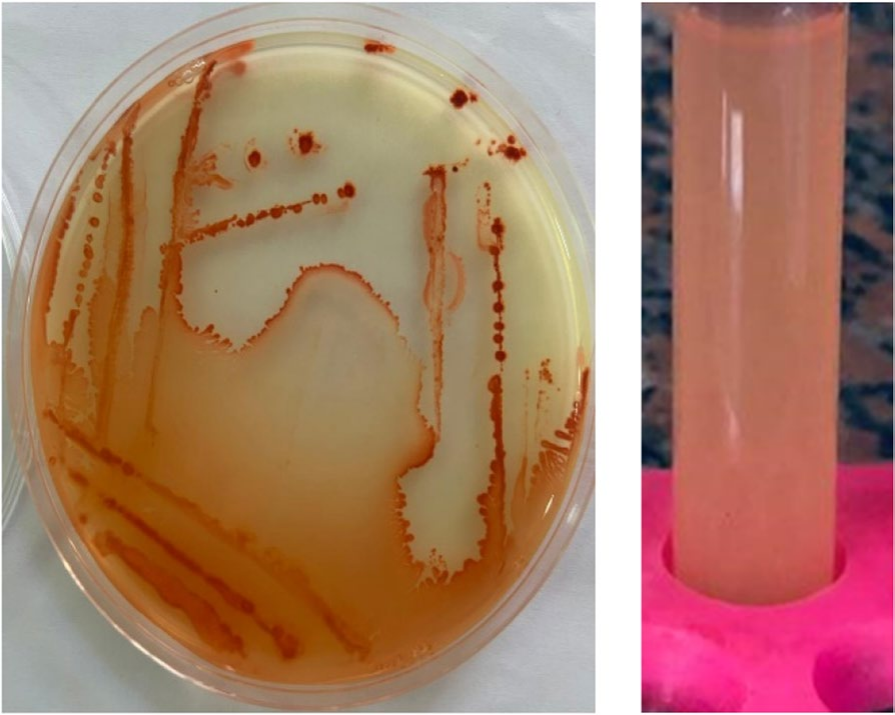
Representative plate and broth showing the growth of selenate reducing *Bacillus cabrialesii* strain Se1 on nutrient medium supplemented with selenate respectively

After enrichment of the collected soil sample from Anani Canal in Al-Merj, Cairo, Egypt (30.15639° N, 31.37110° E) in EBM, five morphologically different orange-red circular and shiny bacterial colonies (Se1, Se2, Se3, Se4, and Se5) were isolated and purified on nutrient agar media supplemented with selenate (Fig. 1).

### Quantitative assay of Se-NPs production

**Fig. 2:**
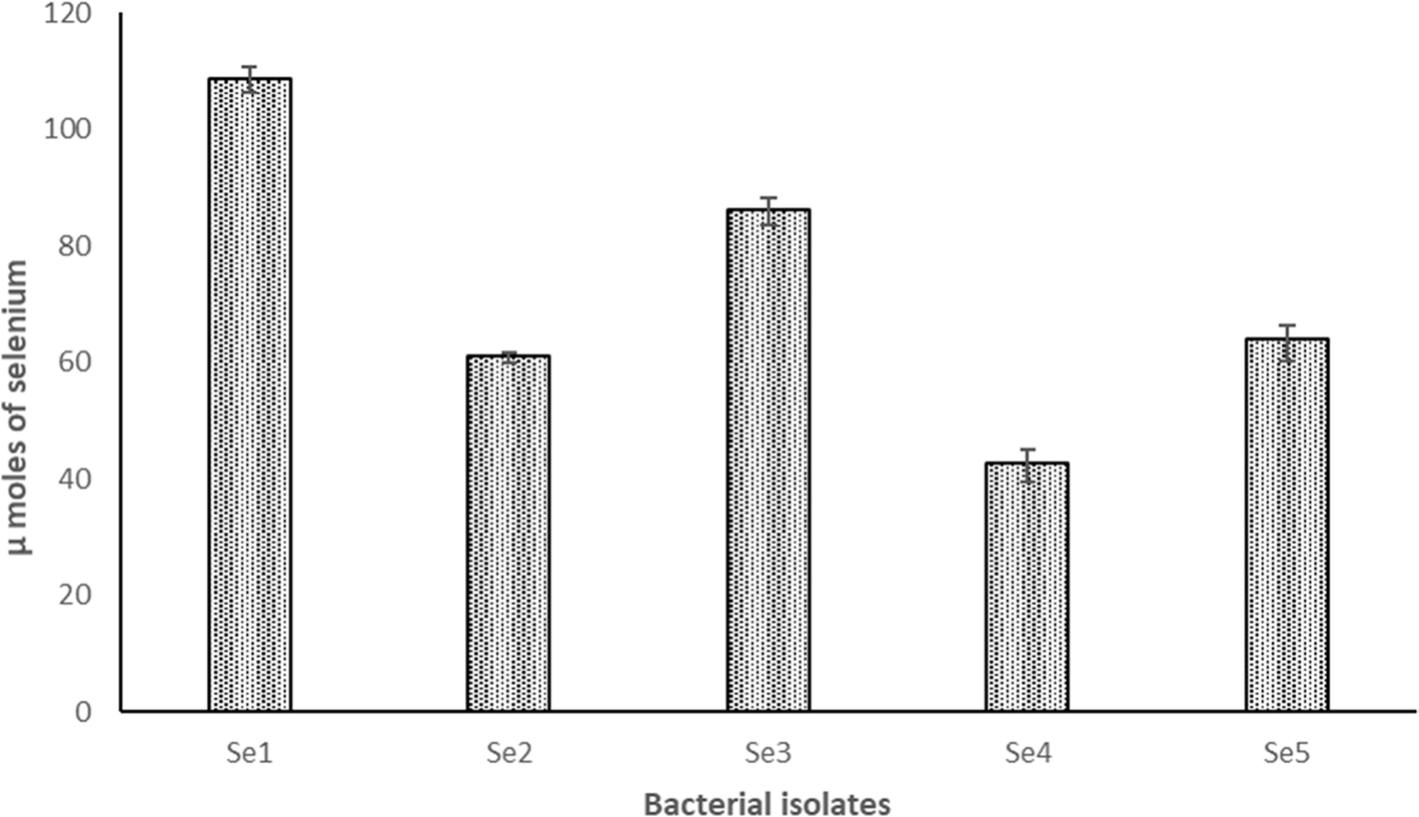
Three bacterial isolates (Se1, Se3 and Se5) showed higher production of Se^0^. The highest amount of Se.^0^ produced by Se1, Se3 and Se5 were 108 ± 1.846, 86.184 ± 1.942 and 63.867 ± 2.483 μmol respectively. All isolates were isolated from Anani Canal in Al-Merj, Cairo, Egypt (30.15639° N, 31.37110° E)

After incubation of bacterial isolates on EBM at 30 °C for 7 days, the bacterial isolates' capacity to reduce selenate (Se^0^ production) and the intensity of red color were measured at 500 nm using a spectrophotometer. The amount of selenium produced by isolates varied between 42.723 ± 2.19 and 108.8 ± 1.846 μmol. The Se1 isolate had the highest percentage of selenate reduction and elemental selenium production (108.8 ± 1.846 μmol) (Fig. 2). The Se1 isolate was selected for further characterization and evaluation of the various conditions influencing selenate reduction and Se-NPs production.

### Characterization of Se-NPs

#### UV-visible absorption spectra

**Fig. 3:**
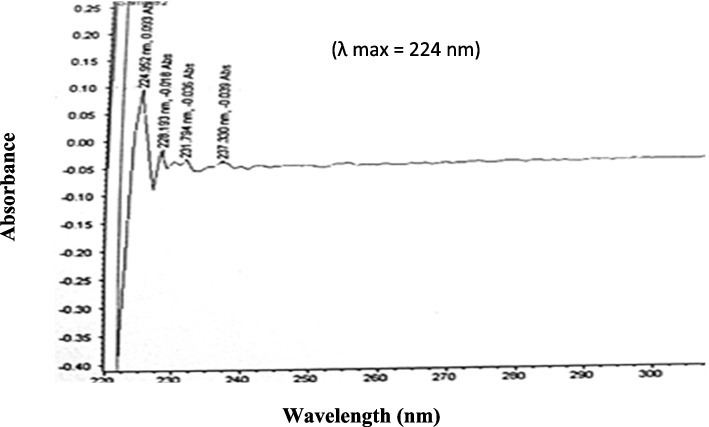
UV–Visible spectrum of Se.^0^ formed by *Bacillus cabrialesii* strain Se1

UV–Vis spectra showed characteristic peaks, indicating the production of elemental selenium. The characteristic absorption bands for Se^0^ were observed at 224 (λ max), 229, and 231 nm, which fall within 200 to 300 nm, indicating that Se-NPs were formed by the reduction of selenate by Se1 isolate (Fig. 3).

### X‑ray diffraction

**Fig. 4:**
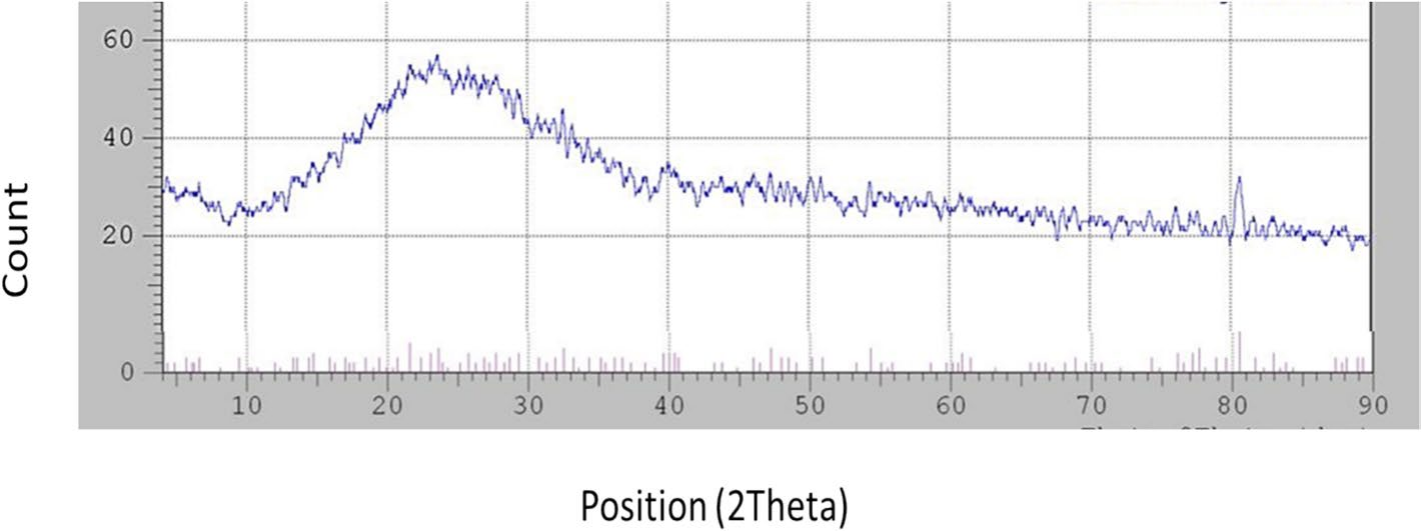
XRD pattern of Se-NPs obtained from *Bacillus cabrialesii* strain Se1

XRD analysis revealed a broad pattern without sharp peaks, indicative of the amorphous nature of the synthesized Se-NPs. Moreover, there were small peaks at 2-theta values of 21.58°, 32.52° and 40.36° (Fig. 4).

### Fourier‑transform infrared spectroscopy

**Fig. 5:**
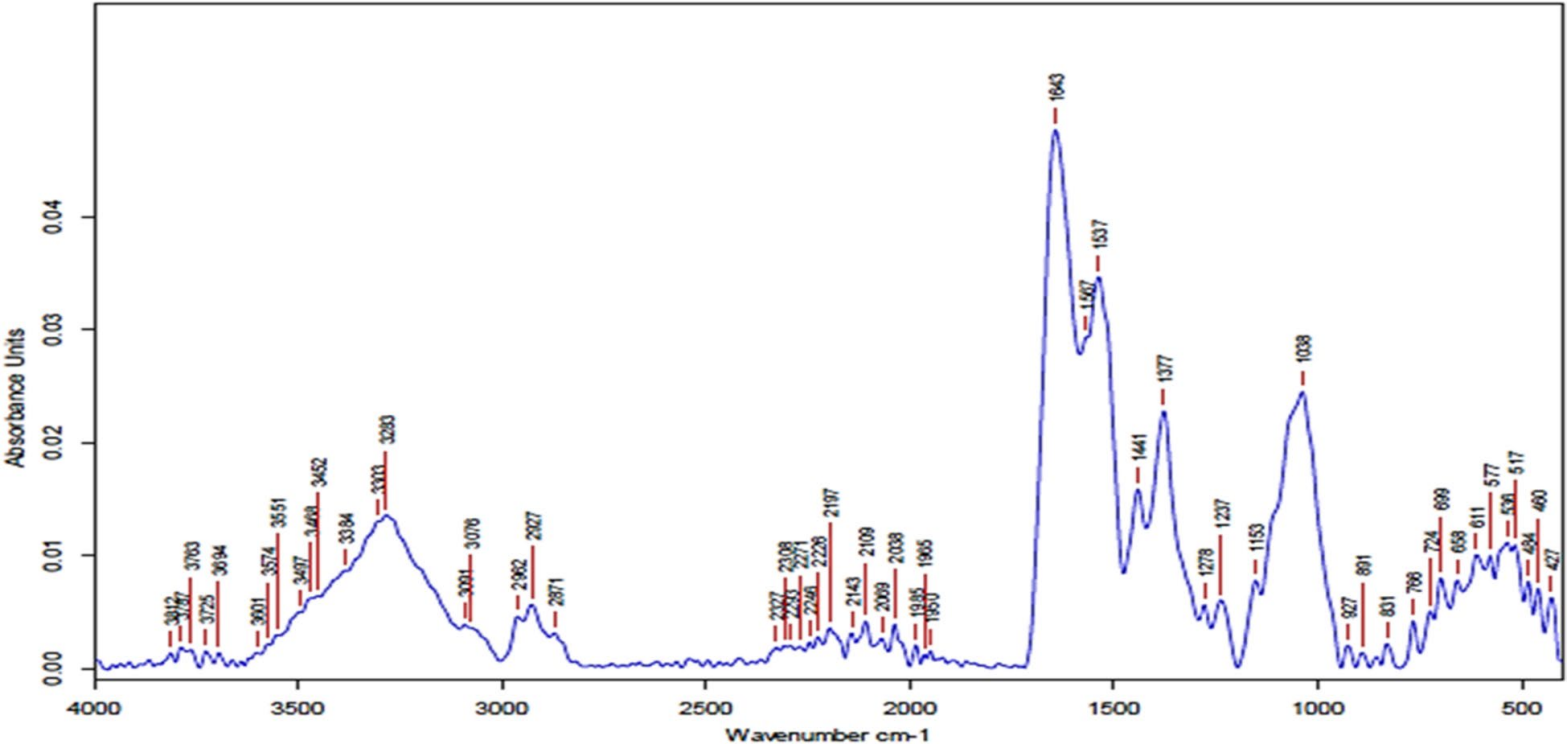
FTIR spectra of Se-NPs produced by *Bacillus cabrialesii* strain Se1

FTIR analysis of synthesized Se-NPs represented a broad band at 3200–3600 cm^⁻1^, indicating O–H bands of hydroxyl groups overlap with N–H stretching of proteins/amine groups. This finding suggests that hydroxyl or amine groups are involved in the capping and stabilization of Se-NPs. Peaks near 2920–2850 cm⁻^1^, representing C–H stretching vibrations (alkyl chains, –CH₂, –CH₃), indicated the presence of lipids, fatty acids, or protein side chains on nanoparticle surfaces. The strong band at ~ 1630–1650 cm⁻^1^ demonstrated the presence of the C = O band of amide I of proteins/peptides bound to Se-NPs, acting as capping/stabilizing agents. At the same time, the bands between 1200 and 1000 cm⁻^1^ of C–O–C stretching (polysaccharides) or C–N stretching (amines) indicated the presence of polysaccharides or proteins around Se-NPs. Bands below 800 cm⁻^1^ represented metal–oxygen or metal–selenium vibrations (M–Se). Moreover, peaks around 500–400 cm⁻^1^ confirmed the formation of Se–Se or Se–O bonds, which are characteristic of Se-NPs (Fig. 5).

### Transmission electron microscope (TEM)

**Fig. 6:**
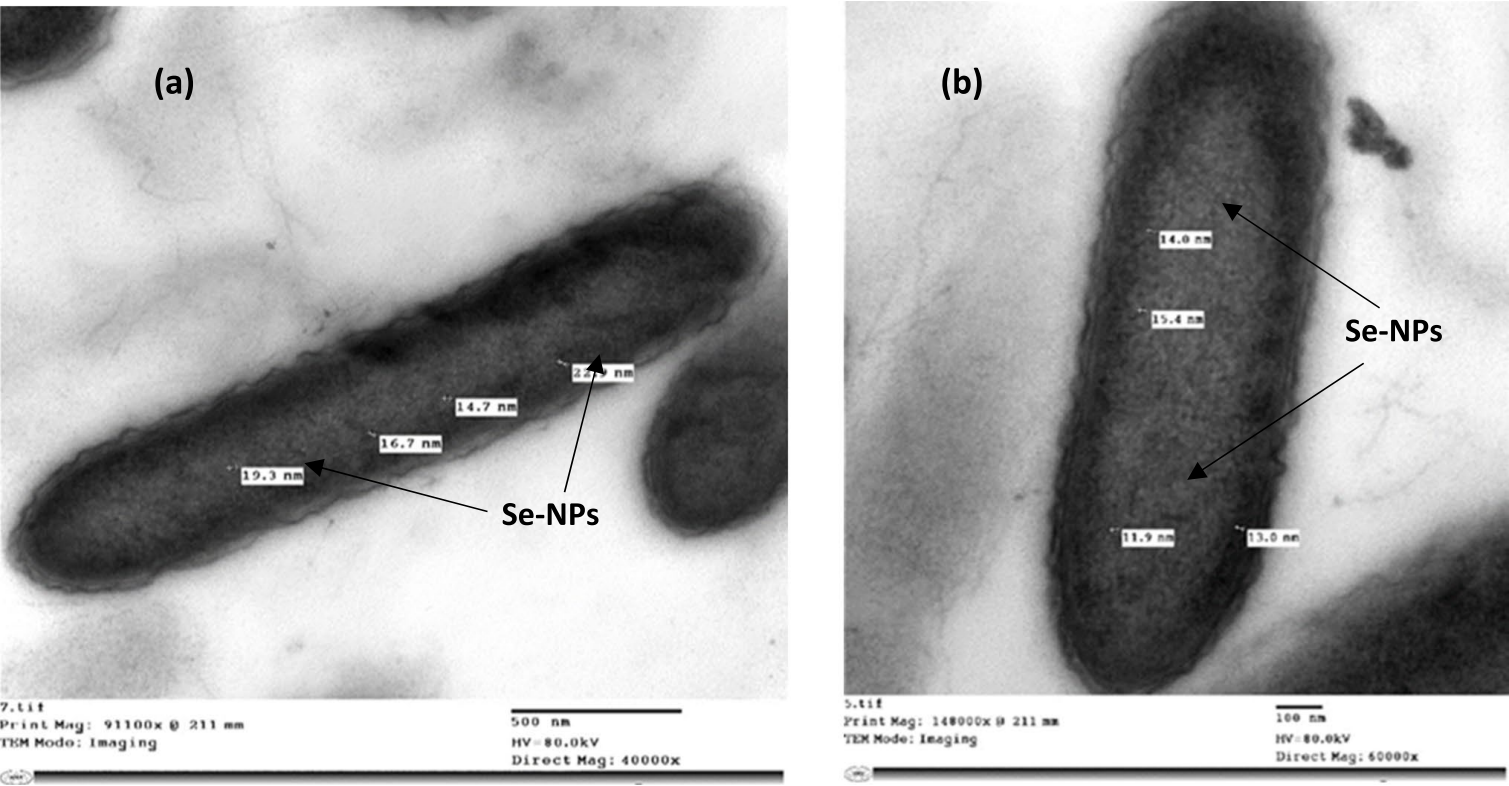
Transmission Electron Micrographs (TEM) of *Bacillus cabrialesii* Se1: **a** and **b**: show Se-NPs associated within the bacterial cells

TEM micrographs of elemental selenium associated with bacterial cells cultured for 24 h at 30 °C on EBM supplemented with 0.0945 gL^−1^ of sodium selenate demonstrated the development of Se-NPs within bacterial cells. The size of Se-NPs associated with the *B. cabrialesii* strain Se1 ranged from 11.9 to 25 nm (Fig. 6).

### Molecular identification of the selenate-reducing bacterial isolate

**Fig. 7:**
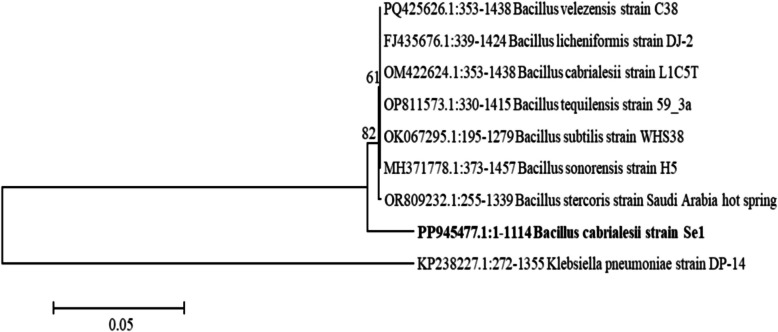
Neighbor-joining phylogenetic tree of 16S rRNA genes. The numbers at the nodes are bootstrap values recovered from 100 trees

The isolate Se1 was identified as *Bacillus cabrialesii* (with a 98% sequence similarity). The nucleotide sequences were submitted to GenBank as *B. cabrialesii* strain Se1 under the accession number PP945477. Notably, to the best of our knowledge, this is the first time *B. cabrialesii* has been shown to be capable of reducing selenate. Phylogenetic analysis of 16S rRNA gene sequences is illustrated in Fig. 7. *B. cabrialesii* strain Se1 was grouped with *Bacillus* strains, confirming the NCBI identification.

### Optimization of selenate reduction by bacterial isolate

The BBD matrix in Table 2 shows 42 experimental runs with different combinations of the received response at both experimental and predicted values and the three uncoded levels of the five variables (A: sodium lactate concentration; B: medium pH; C: incubation temperature; D: incubation period; E: sodium selenate concentration).

**Table 2:** The Box-Behnken design (BBD) matrix by the five tested variables in addition the observed vs. predicted values by selected isolate

Run	Point Type	**Blocks**	Coded parameters	**B**	**C**	**D**	**E**		Mean Se^0^ concentration (μmol)		
			**A**	**B**	**C**	**D**	**E**		**Observed**	**Predicted**	
1	2	1	0.5	6	30	6	15		34.72	39.64	
2	2	1	5	6	30	6	15		54.95	61.24	
3	2	1	0.5	9	30	6	15		65.8	66.39	
4	2	1	5	9	30	6	15		70.41	72.37	
5	2	1	2.75	7.5	25	2	15		60.8	65.79	
6	2	1	2.75	7.5	35	2	15		35.95	39.54	
7	2	1	2.75	7.5	25	10	15		85.56	92.89	
8	2	1	2.75	7.5	35	10	15		104.8	110.73	
9	2	1	2.75	6	30	6	5		55.64	52.14	
10	2	1	2.75	9	30	6	5		95.03	88.05	
11	2	1	2.75	6	30	6	25		48.87	47.68	
12	2	1	2.75	9	30	6	25		54.33	49.66	
13	2	1	0.5	7.5	25	6	15		65.03	52.60	
14	2	1	5	7.5	25	6	15		77.72	68.85	
15	2	1	0.5	7.5	35	6	15		66.64	50.86	
16	2	1	5	7.5	35	6	15		74.41	62.19	
17	2	1	2.75	7.5	30	2	5		53.1	49.29	
18	2	1	2.75	7.5	30	10	5		155.83	141.15	
19	2	1	2.75	7.5	30	2	25		66.26	70.59	
20	2	1	2.75	7.5	30	10	25		83.56	77.01	
21	2	1	2.75	6	25	6	15		44.26	43.39	
22	2	1	2.75	9	25	6	15		59.03	65.03	
23	2	1	2.75	6	35	6	15		40.49	41.89	
24	2	1	2.75	9	35	6	15		49.87	58.13	
25	2	1	0.5	7.5	30	2	15		58.3	60.89	
26	2	1	5	7.5	30	2	15		59.18	60.05	
27	2	1	0.5	7.5	30	10	15		90.72	95.40	
28	2	1	5	7.5	30	10	15		120.87	123.82	
29	2	1	2.75	7.5	25	6	5		63.87	65.41	
30	2	1	2.75	7.5	35	6	5		68.18	72.20	
31	2	1	2.75	7.5	25	6	25		52.72	54.99	
32	2	1	2.75	7.5	35	6	25		35.03	39.78	
33	2	1	0.5	7.5	30	6	5		47.1	60.39	
34	2	1	5	7.5	30	6	5		82.74	92.82	
35	2	1	0.5	7.5	30	6	25		55.49	57.60	
36	2	1	5	7.5	30	6	25		53.87	52.77	
37	2	1	2.75	6	30	2	15		49.33	42.56	
38	2	1	2.75	9	30	2	15		71.18	65.35	
39	2	1	2.75	6	30	10	15		95.87	95.55	
40	2	1	2.75	9	30	10	15		110.03	110.65	
41	0	1	2.75	7.5	30	6	15		123.876	117.91	
42	0	1	2.75	7.5	30	6	15		111.953	117.91	

*Bacillus cabrialesii* strain Se1 was inoculated in EBM supplemented with sodium selenate. Runs were duplicated, and the mean Se^0^ concentrations was calculated

**Fig. 8:**
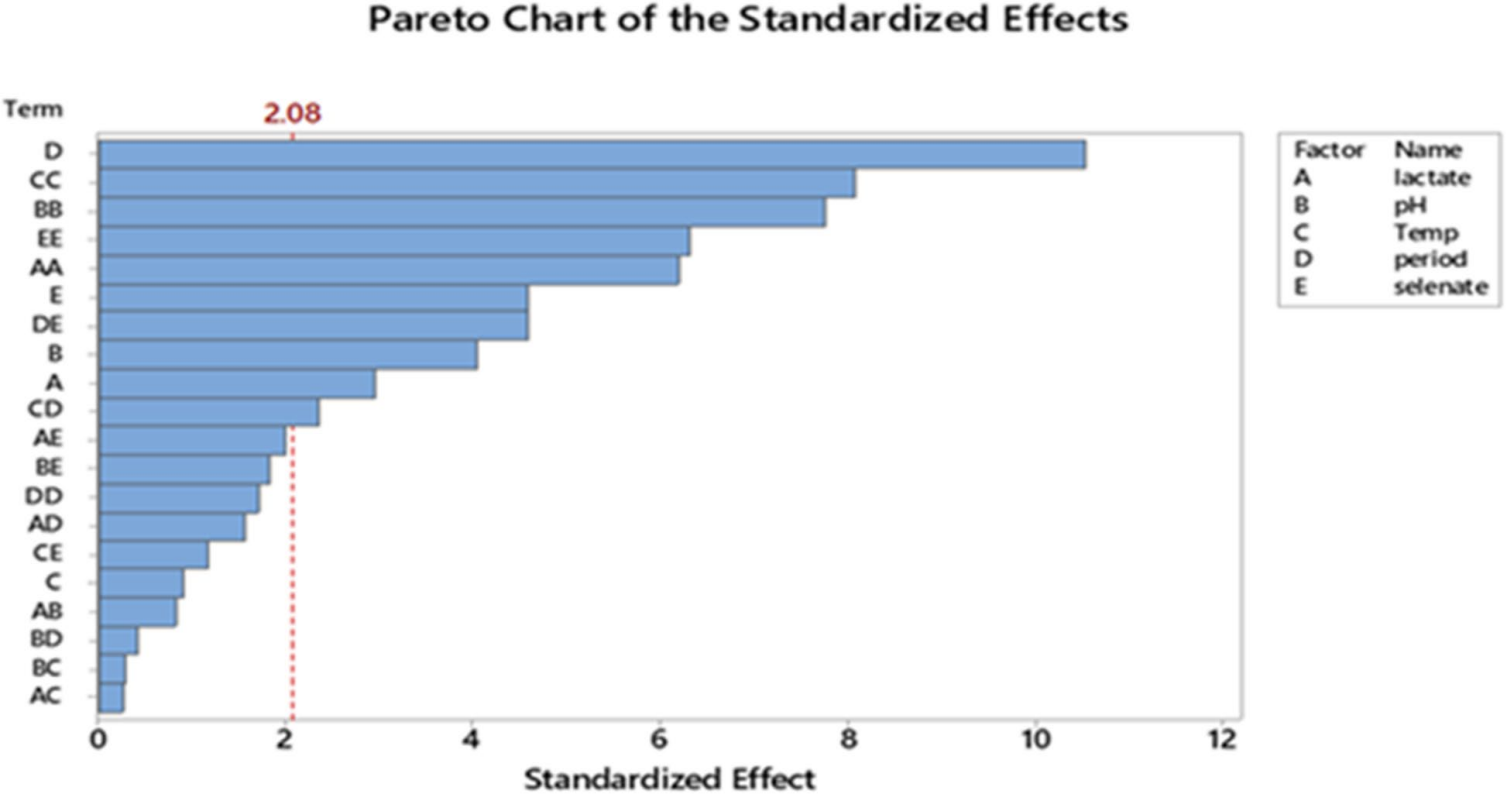
Pareto chart of the standardized effects of the five factors, response was Se^0^ yield,* P* < 0.05

**Fig. 9:**
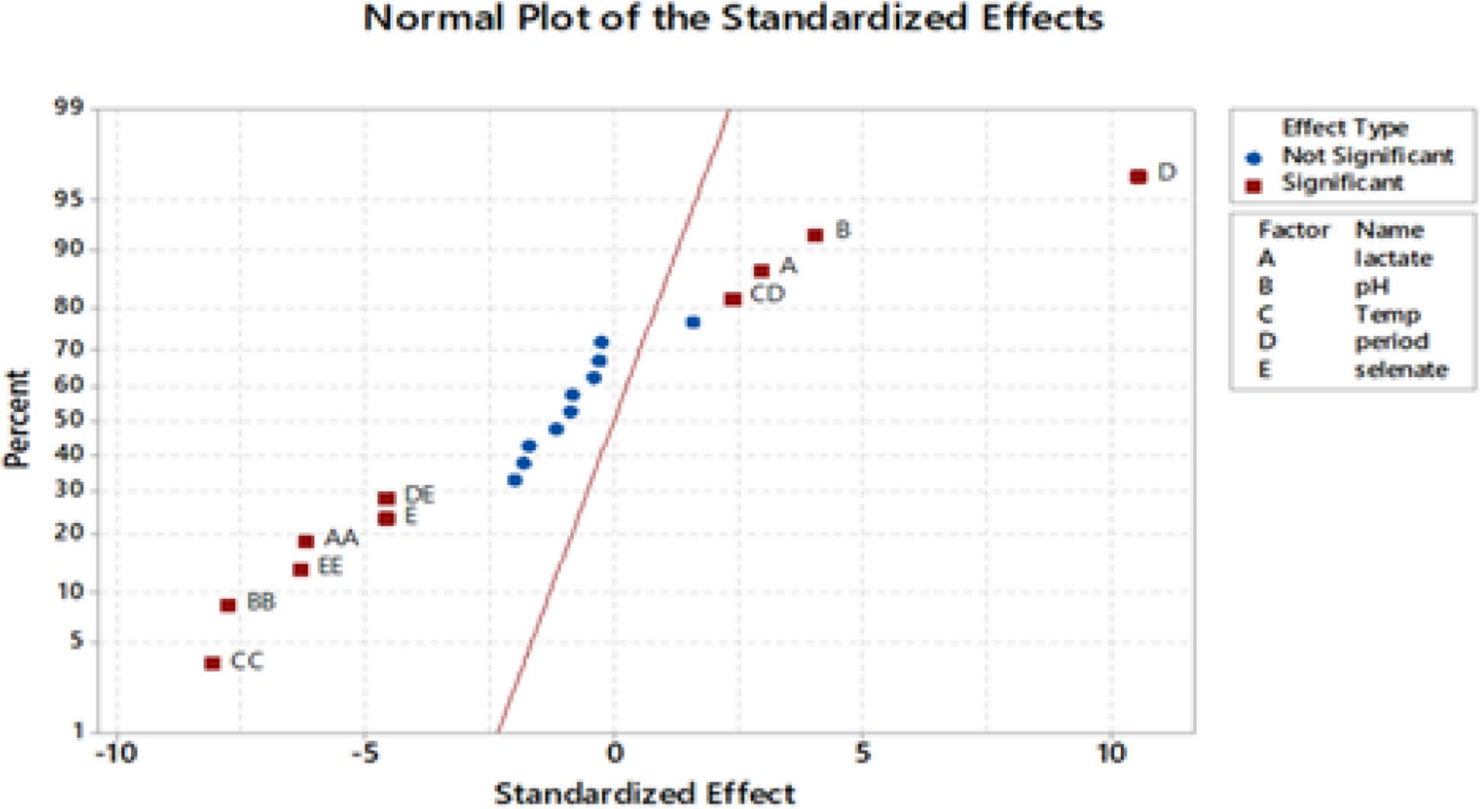
Normal plot of the standardized effects, response is Se^0^ yield, *P* < 0.05

**Fig. 10:**
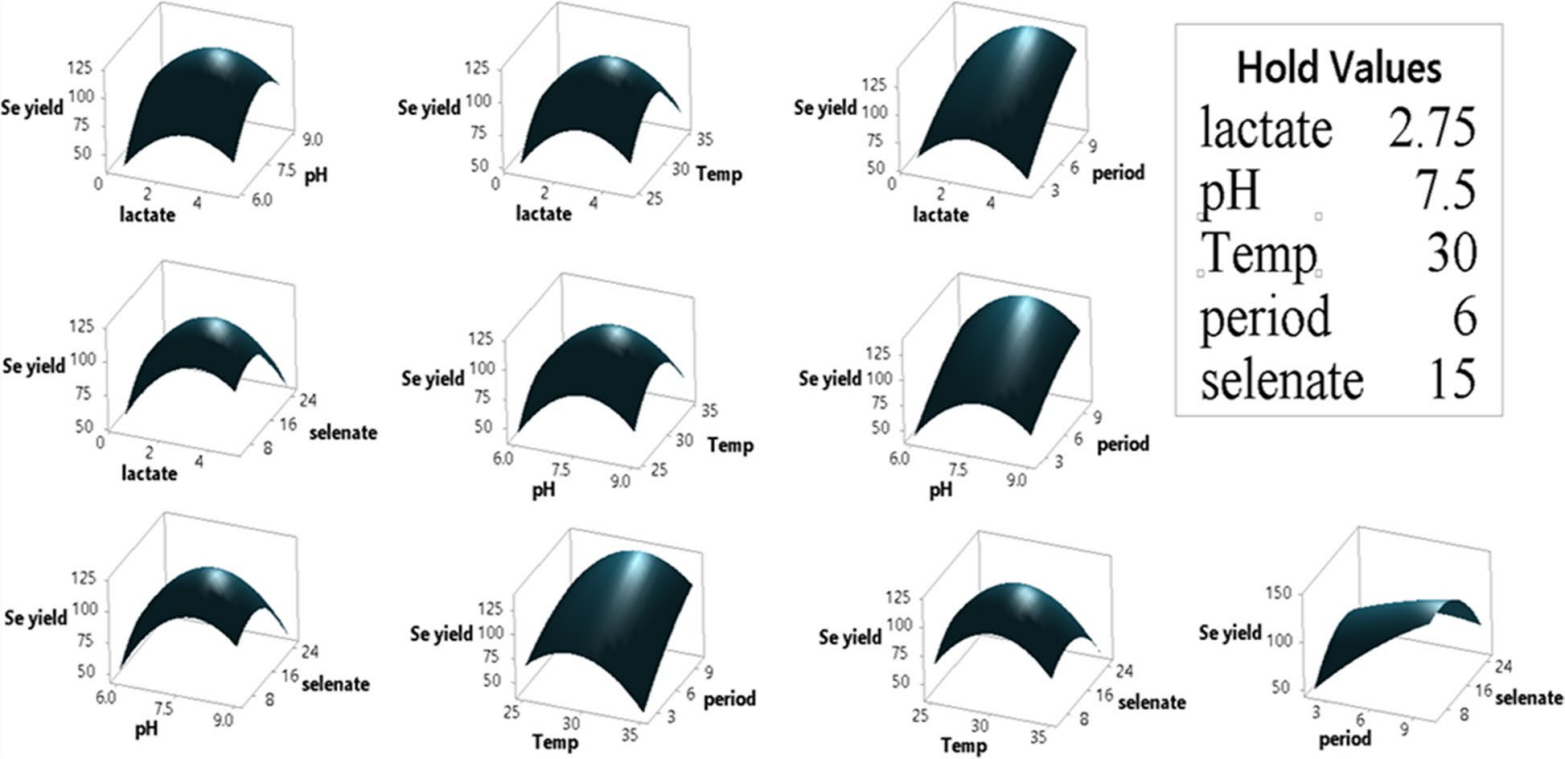
The 3D surface plots show the interaction effect and the optimum levels of thesignificant factors on selenate reduction by *Bacillus cabrialesii* strain Se1

As demonstrated, the assessed selenate concentration varied between 5 gL^−1^ and 25 gL^−1^, indicating a significant (*p*-value < 0.05) effect of the process parameters under investigation on selenate reduction. The ANOVA for the BBD data under study showed that the model resulted in a significant difference (model *p*-value < 0.05) in selenate reduction. According to Shubharani et al. [59], the model's high F-value and low *p*-values (*p* < 0.05) indicate a highly accurate prediction for the response [21]. After analyzing the main, squared, and interaction impacts of the chosen factors, the following variables were found to have a significant effect on selenate reduction and Se^0^ production (*p*-value < 0.05) because the response surface regression analysis for the BBD was substantial (*p*-value < 0.05): A, AA, B, BB, C. CC, E, EE, DE, and CD, as shown in the ANOVA table and the graphical representation of the Pareto chart (Fig. 8). Furthermore, as shown in the normal plot (Fig. 9) and coded coefficients in Table 3, positive T-values demonstrate a synergistic effect of the various factors under study and their interactions on the selenate decrease and Se^0^ production, whereas negative T-values indicate an antagonistic effect [3]. Consequently, the selenate reduction process by *Bacillus cabrialesii* strain Se1 was positively and significantly (*p*-value < 0.05) influenced by the individual factors of initial medium pH (Factor B) at a low level of 7.7 and incubation temperature (Factor C) at a low level of 30°C. Additionally, both factors reveal a significantly positive interaction effect (*p*-value of AB < 0.05). Stated differently, a high synergistic impact on selenate reduction was achieved with an initial medium pH of 6 to 9 and an incubation temperature of 25 °C to 35°C. Notably, the incubation period (Factor D) at a level of 10 days and the initial selenate concentration (Factor E) at a level of 5 to 25 gL^−1^ had a significantly positive interaction effect (*p*-value < 0.05) on selenate bioreduction by the bacterial strain under study (Ghasemi et al. 2009 [23]; [37]). Thus, the interactions between the selected variables under investigation and the desired response were successfully identified by the statistical model used (Llamosas et al. 2020)[36]. Furthermore, all possible interactions between the variables under investigation and their levels were graphically represented using 3D surface plots (Fig. 10).

**Table 3:** Coded Coefficients for the linear, quadratic, and interaction terms of the applied BBD that describes the relationship between independent variables and the response (Se^0^ yield)

Term	Coef	SE Coef	T-Value	*P*-Value	VIF
Constant	117.91	6.60	17.87	0.000	
Lactate, gL^−1^	6.90	2.33	2.96	0.008	1.00
pH	9.47	2.33	4.06	0.001	1.00
Temp, °C	−2.10	2.33	−0.90	0.378	1.00
period, h	24.57	2.33	10.53	0.000	1.00
Selenate, gL^−1^	−10.71	2.33	−4.59	0.000	1.00
(Lactate, gL^−1^)^2^	−25.74	4.15	−6.20	0.000	1.96
(pH)^2^	−32.26	4.15	−7.77	0.000	1.96
(Temp, °C)^2^	−33.54	4.15	−8.08	0.000	1.96
(period, h)^2^	−7.13	4.15	−1.72	0.101	1.96
(Selenate, gL^−1^)^2^	−26.27	4.15	−6.33	0.000	1.96
Lactate × pH	−3.90	4.67	−0.84	0.412	1.00
Lactate × Temp	−1.23	4.67	−0.26	0.795	1.00
Lactate × period	7.32	4.67	1.57	0.132	1.00
Lactate × selenate	−9.31	4.67	−2.00	0.059	1.00
pH × Temp	−1.35	4.67	−0.29	0.776	1.00
pH × period	−1.92	4.67	−0.41	0.685	1.00
pH × selenate	−8.48	4.67	−1.82	0.083	1.00
Temp × period	11.02	4.67	2.36	0.028	1.00
Temp × selenate	−5.50	4.67	−1.18	0.252	1.00
Period × selenate	−21.36	4.67	−4.58	0.000	1.00

**Fig. 11:**
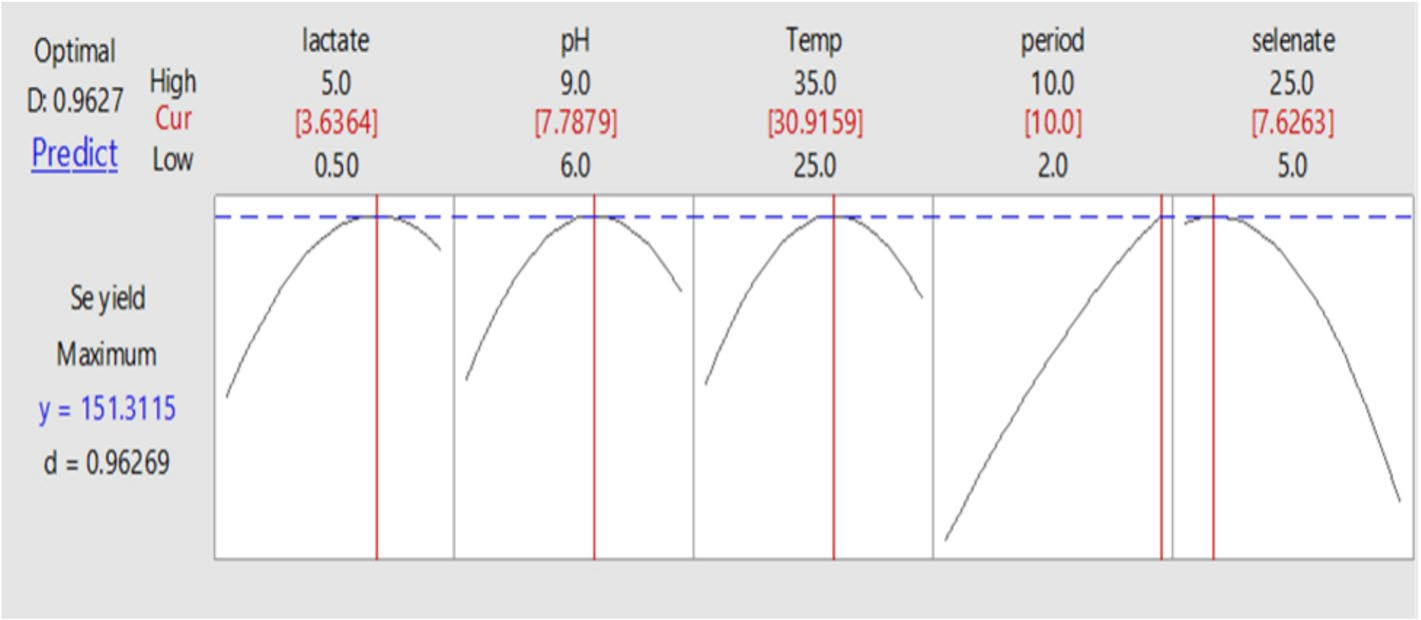
Predicted solution for the maximum selenate reduction using the response optimizer of Minitab Software v. 18 defining the optimum levels of the selected five variables, growth medium pH, temperature, incubation period, selenate concentration and sodium lactate concentration. (Cur) is the curvature value (optimum), y is the maximum selenium Se.^0^ production predicted and D is the desirability value by *Bacillus cabrialesii* strain Se1

While maintaining the other parameters at their central values, the interactive impacts between each pair of parameters were demonstrated. The results of the response optimizer (Fig. 11) demonstrated that *Bacillus cabrialesii* strain Se1 could achieve a maximum production of Se-NPs (151.311 μmol) at a medium pH of 7.8, a temperature of 31 °C, an incubation period of 10 days, a sodium selenate concentration of 7.6 gL^−1^, and a sodium lactate concentration of 3.6 gL^−1^ with a desirability of 0.96269. The validation experiment was conducted in triplicate to confirm these predicted values, and a maximum mean observed Se0 production of 153.75 ± 1.2 μmol was effectively achieved, which was very close to the expected yield of 151.311 μmol, as anticipated by the applied statistical model.

### Regression equation in uncoded units

Se yield (μmol) =—2227 + 44.3 (lactate, gL^−1^) + 240.3 (pH) + 80.1 (Temp, °C) + 3.13 (period, h) + 18.69 (selenate, gL^−1^)—5.085 (lactate, gL^−1^)^2^—14.34 (pH)^2^—1.342 (Temp, °C)^2^—0.445 (period, h)^2^—0.2627 (selenate, g L^−1^)^2^—1.16 (lactate × pH)—0.109 (lactate × Temp) + 0.813 (lactate × period)—0.414 (lactate × selenate)—0.180 (pH × Temp)—0.320 (pH × period)—0.565 (pH × selenate) + 0.551 (Temp × period)—0.1100 (Temp × selenate)—0.534 (period × selenate).

### Antimicrobial effect of selenium nanoparticles produced by B. cabrialesii strain Se1

**Fig. 12:**
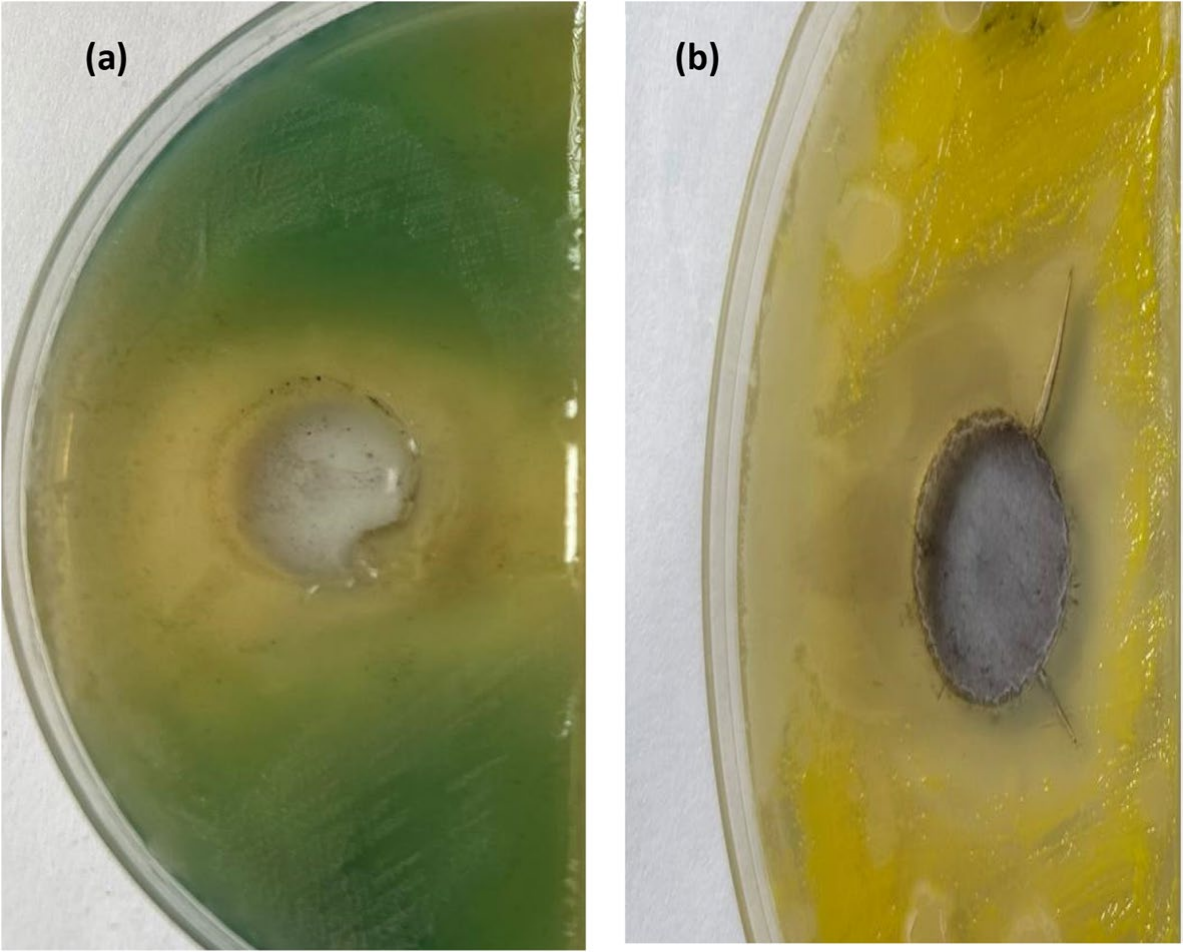
The antibacterial effect of synthesized selenium nanoparticles (Se-NPs) on *P. aeruginosa* (**a**) and *S. aureus* (**b**)

Se-NPs produced by *B. cabrialesii* strain Se1 showed potential preliminary antibacterial activity against two pathogens, *Staphylococcus aureus* and *Pseudomonas aeruginosa*, with inhibition zones of 12.5 ± 0.3 and 20 ± 0.08 mm, respectively (Fig. 12).

## Discussion

The increasing industrial and agricultural use of selenium is expected to release larger amounts of its toxic forms, particularly selenate and selenite, into the environment, posing significant risks to both terrestrial and aquatic ecosystems. Currently, only about 15% of approximately 2,700 tons of selenium produced annually is recycled (Seyedali et al. 2021)[66]. Selenium’s high biological, photoelectric, and semiconductor qualities make it useful in a wide range of sectors. Selenium has been effectively used in solar cells, rectifiers, photographic exposure meters, and xerography (Gates et al. 2005[24]; [29]).

Selenium is an essential trace element required for maintaining physiological balance in both humans and animals. It possesses potent antibacterial, anti-inflammatory, and antioxidant properties. Selenium primarily exerts its biological functions through its incorporation into selenoproteins, which contain the amino acid selenocysteine [49, 53]. To prevent selenium deficiency—which can lead to health problems affecting multiple organs and systems—dietary supplementation is commonly used in both organic forms (selenomethionine and selenocysteine) and inorganic forms (selenate and selenite) [26, 40]. In animal production, selenium supplementation has been applied to improve meat quality, immune response, and growth performance in pigs and other livestock [4, 31, 67, 68].

A key process for removing selenium from aquatic environments involves microbes reducing soluble selenium oxyanions, such as selenate and selenite, to elemental selenium, which then accumulates in sediments [31, 51]. Several microorganisms were identified as selenate-reducing bacteria, including *Acinetobacter* [7], *Aeromonas* [7, 14], *Arthrobacter* [7], *Bacillus* [7, 57]; Sarathchandra et al. 1984 [69]), *Candida* [28], *Cephalosporium* [8], *Citrobacter* [7], *Corynebacterium* [7], and *Flavobacterium* [7], with additional reports by Tabbene et al. [74], Chung et al. [10], and Fiddaman et al. [20].

In this study, a polluted soil sample was collected, enriched on EBM, and then subcultured on a nutrient agar medium supplemented with sodium selenate (0.0945 gL^−1^) to isolate selenate-reducing bacteria, following a method similar to that of Zhang et al. [80]. Five distinct bacterial isolates (Se1, Se2, Se3, Se4, and Se5) with orange/red colonies were obtained, indicating their ability to convert selenate to elemental selenium. The intensity of the red color, measured spectrophotometrically at 500 nm, correlated with the efficiency of selenate reduction, consistent with findings by Gates et al. (2005) [24] and Wellen et al. [75].

Isolate Se1, sourced from Anani Canal in Al-Merj, Cairo, Egypt (30.15639° N, 31.37110° E), exhibited the highest selenium production capability, yielding 108.8 ± 1.846 μmol.

The synthesized Se-NPs were characterized using UV–Vis spectroscopy, revealing characteristic peaks indicative of elemental selenium production. The absorption bands for Se⁰ were observed at 224, 229, and 231 nm, within the 200–300 nm range, confirming the formation of Se-NPs by reducing selenate using the Se1 isolate. This finding is consistent with previous studies [32, 39] that reported maximum absorption bands between 200 and 300 nm for cultures containing Se^0^.

The XRD pattern indicated the amorphous nature of the synthesized Se-NPs by the selected isolate. This finding is comparable to those of Rajkumar et al. [56] and Chandramohan et al. [15], who synthesized Se-NPs using various bacterial strains.

The FTIR spectrum confirms that the Se-NPs are surrounded by biomolecules, such as proteins and polysaccharides, which act as reducing, capping, and stabilizing agents, as indicated by the peaks at 3200–3600, 2920–2850, 1630–1650, 1038, 1237, and 1278 cm⁻^1^. The peaks at lower wavenumbers (800, 400–500 cm^−1^) confirm the presence of Se–O/Se–Se vibrations, supporting the formation of Se-NPs. The 400–1500 cm^⁻1^ region highlighted a unique fingerprint that allows for the detection of subtle differences between molecules [48]. This was expected and parallel to the finding of Chandramohan et al. [15], who reported that the absorption broad peaks of Se-NPs were observed at 1670, 1516, and 420 cm⁻^1^, corresponding to reducing groups (C = O, NH, C = C) involved in Se-NPs formation. The FTIR spectrum of the selenium showed bands observed at 3441, 2920, 2858, 1625, 1537, 1324, 1025, and 1032 cm^−1^. These are associated with O–H stretch, free hydroxyl-C-H stretch, H-C-H stretch = C-H asymmetric stretch = O bend, and C-O stretch. The strong absorption bands at 1649 and 1551 cm-1 are characteristic of the amide I and C-H vibrations of the CH2 groups of the protein moiety, respectively, with albumin serving as the stabilizing and capping agent surrounding the Se-NPs [54]. Saravanan et al. [64] demonstrated that FT-IR analysis revealed interactions between selenium and metabolites, facilitating the synthesis of Se-NPs by serving as both capping and reducing agents. Peaks at 743.39 cm⁻^1^ (C–H bending, aromatic compounds) and 1258.98 cm⁻^1^ (C–O stretching, alcohols/ethers) indicate the presence of reducing and capping agents. Moreover, a peak at 1290 cm⁻^1^ is associated with C = O and -NH2 functional groups as reducing agents for Se-NPs production.

The formation and size of red Se-NPs produced via sodium selenate reduction were analyzed using TEM. *Bacillus cabrialesii* strain Se1 demonstrated internal Se-NPs, aligning with the findings of Meyer et al. [46], who reported the synthesis of Se-NPs through selenate reduction by *Acinetobacter* species.

In this study, a BBD approach was employed to investigate the various factors influencing microbial selenate reduction and Se-NP production. These factors included sodium lactate concentration, pH, incubation temperature, incubation time, and sodium selenate concentration. BBD offers several advantages, including the ability to predict maximum information with minimal experimental trials, assess the nonlinearity of dependent variables, and reduce the number of experiments required to estimate quadratic terms in a second-order model.

The maximum concentration of Se-NPs produced was 151.311 μmol. The optimal sodium lactate concentration required for effective selenate reduction and maximum Se-NPs yield was determined to be 3.6 gL^−1^. This finding aligns with previous studies that reported an optimal lactate concentration range of 0.5 to 5.0 gL^−1^ [21, 70, 71]. The ideal pH for achieving the maximum Se-NPs production (151.311 μmol) was 7.8, which is consistent with Salem et al. [70], who reported an optimal pH of 7.5. Other previous studies suggested a broader optimal pH range between 6.0 and 9.0 [21, 33, 71]. The optimal temperature for maximum Se-NPs production was 31 °C, which is similar to the values reported by Fahmy et al. [21] and Singh et al. [71], who identified 25 °C and 35 °C, respectively, as ideal temperatures for elemental selenium production. The highest Se-NPs concentration was achieved after a 10-day incubation period. In contrast, Kumar et al. [33] reported five days as the ideal incubation time for selenium nanoparticle synthesis. Lastly, the optimum sodium selenate concentration for maximum Se-NPs production was found to be 7.6 gL^−1^. This value differs slightly from previous reports, where Fahmy et al. [21] identified 5 gL^−1^ as optimal for selenate reduction, while Singh et al. [71] reported 20 gL^−1^ as ideal for elemental selenium production.

In the present study, the strain Se1 obtained was identified through 16S rRNA gene sequencing as *Bacillus cabrialesii* strain Se1 (GenBank accession no. PP945477). Overall, sequence divergence within the 16S rRNA gene of the genus *Bacillus* has been shown to be generally sufficient for resolving most species [9, 82]. Furthermore, comparative assessments of 16S rRNA sub-regions indicated that the V4–V6 regions provide the most reliable representation of full-length sequences in phylogenetic analyses across bacterial phyla [82]. In the context of the present study, the sequenced 16S rRNA fragment (~ 1114 bp) encompassed both the V4-V6 hypervariable regions, thereby strengthening the reliability of this taxonomic identification. To the best of our knowledge, this is the first report identifying *Bacillus cabrialesii* as a selenate-reducing bacterium. *B. cabrialesii* is also recognized for its plant growth-promoting properties and its role as a biological control agent in wheat cultivation [41, 45].

Selenate reduction results in the formation of elemental selenium or Se-NPs, which appear as small reddish deposits. Se-NPs have demonstrated significant antibacterial potential due to their strong adsorptive and biological properties, attributed to interactions with various protein functional groups such as C–O, C–N, NH, and COO⁻ [77, 78]. Additionally, Yang et al. [77, 78] evaluated the antimicrobial efficacy of selenium-conjugated nanoparticles combined with quercetin and cholinergic agents against multidrug-resistant pathogens, including *Escherichia coli* and *Staphylococcus aureus*, highlighting their potential in treating infectious diseases. In the same study, Se-NPs were shown to inhibit *Staphylococcus* biofilm formation by up to 60-fold. The antibacterial action of Se-NPs is primarily due to their ability to penetrate bacterial membranes, leading to membrane disruption, lysis, and ultimately, cell death. These are among the key mechanisms by which Se-NPs exert their antimicrobial effects. Furthermore, Se-NPs produced by *Bacillus cabrialesii* strain Se1 exhibited promising antimicrobial activity against the pathogens *Staphylococcus aureus* and *Pseudomonas aeruginosa*. These results agree with the findings of Yang et al. [77, 78] and Kumar et al. [34], who reported the effectiveness of Se-NPs against various pathogens, including *Escherichia coli*, *Pseudomonas* species, and *Staphylococcus aureus*.

## Conclusion

The strain *Bacillus cabrialesii* Se1 was isolated from polluted soil from Anani Canal in Al-Merj, Cairo, Egypt (30.15639° N, 31.37110° E) and recorded as a selenate-reducing bacterium for the first time. The isolated bacteria were enriched on EBM and then measured to determine the capacity to reduce selenate and the intensity of red color using a spectrophotometer at 500 nm, showing the highest production of Se-NPs (Se1) (108.8 ± 1.846 μmol). Se^0^ formation was confirmed and characterized by the characteristic peaks at 224, 229, and 231 nm obtained from UV–Vis spectra. Se-NPs were detectable within the bacterial cells upon examination using TEM, with a size ranging from 11.9 to 25 nm. Using the BBD, the reduction of sodium selenate by *B. cabrialesii* strain Se1 was optimized with a sodium lactate concentration of 3.6 gL^−1^, a medium pH of 7.8, a temperature of 31 °C, a 10-day incubation period, and a sodium selenate concentration of 7.6 g L^−1^. With these settings, maximum Se^0^ production (151.311 μmol) was achieved. The strain is a suitable candidate for in situ bioremediation, according to the experimental data in this investigation. 16S rRNA gene sequencing was used to identify the isolate and was submitted to GenBank as *Bacillus cabrialesii* strain Se1 (PP945477). In conclusion, Se^0^ nanoparticles demonstrated high antibacterial activity against two pathogens.

## Data Availability

The NCBI accession number for the 16S rRNA gene sequence of strain *Bacillus cabrialesii* Se1 is PP945477.
